# Determination of Multimycotoxin in Cereal-Based Products Sold in Open-Air Markets

**DOI:** 10.3390/foods12142744

**Published:** 2023-07-19

**Authors:** Funda Yilmaz Eker, Karlo Muratoglu, Muhsin Ozturk, Bayram Cetin, Serkan Kemal Buyukunal

**Affiliations:** 1 Department of Food Hygiene and Technology, Faculty of Veterinary Medicine, İstanbul University-Cerrahpaşa, Avcılar, İstanbul 34320, Türkiyeserkanbuyukunal@iuc.edu.tr (S.K.B.); 2 Department of Gastronomy and Culinary Arts, School of Applied Sciences, İstanbul Esenyurt University, Esenyurt, İstanbul 34510, Türkiye; muhsinozturk@esenyurt.edu.tr; 3 Department of Food Engineering, Engineering Faculty, Kırklareli University, Kırklareli 39000, Türkiye; bayram.cetin@klu.edu.tr

**Keywords:** grain, mycotoxin, aflatoxin, fumonisin, LC/MS-MS, co-occurrence

## Abstract

In this study, a total of 140 cereal-based foods sold in temporary open-air markets were analyzed by LC-MS/MS for aflatoxin B_1_, B_2_, G_1_, G_2_, ochratoxin (OTA), zearalenone (ZEN), deoxynivalenol (DON), fumonisin B_1_, fumonisin B_2_, citrinin (CIT), HT-2, and T-2 toxins. Breakfast cereals (n:27), cornmeal (n:41), extruded maize (n:32), and oatmeal (n:40) purchased from these alternative shopping areas created to meet the food needs of low-income people in the suburbs formed the sample set of the study. These foods, which are sold in areas that are out of legal control and greatly affected by external environmental conditions, are more open to health risks. Mycotoxins, chemicals of a biological origin, are some of the most important of these risks. In terms of public health, it is important to investigate the presence of mycotoxins in foods, which can cause acute and chronic diseases such as immunosuppression, genotoxic, estrogenic, teratogenic effect, cancer, and liver and kidney dysfunctions. Grain-based foods are often contaminated with a large number of mycotoxins, but legal regulations have not been prepared that consider the health risks associated with the co-existence of mycotoxins. Many of the studies have focused on the presence of a single mycotoxin and the risks it poses. As a result, aflatoxin B_1_ levels in 28.57% of the samples and total aflatoxin (B_1_ + B_2_ + G_1_ + G_2_) levels in 26.43% of the samples were determined to exceed the limits defined in the “Turkish Food Codex Contaminants Regulation”. Citrinin could not be detected in any of the samples. The rate of mycotoxin occurrences above the limit of detection (LOD) in grain-based food samples ranged from 22.86% to 99.29%. Total aflatoxin (TAF) + Total Fumonisin (FUM) were found in 83.57% of the samples; TAF + FUM + OTA in 82.14%; TAF + FUM + OTA + T-2 in 44.29%; TAF + FUM + OTA + DON + HT-2, TAF + FUM + OTA + DON + T-2, and TAF + FUM + OTA + DON + ZEN in 22.86% of the samples.

## 1. Introduction

Today, the demographic structure of cities is changing with intense internal and external migration. Demands vary depending on culture, habits, and income level of people in cities with increasing populations. Alternative shopping areas are created in cities, especially in suburbs, to meet the food needs of people of different socio-economic levels [1]. Open-air markets are one of these alternatives. These are temporary outdoor marketplaces on private property where individual vendors offer produce, food, or other products for sale, directly to the consumer. In these areas, which are out of legal control and greatly affected by external environmental conditions, the possible public health risks of the foods offered for sale are also increasing [2]. Cereals and cereal-based products such as cornmeal, extruded maize snacks, maize-based breakfast cereals, and oatmeal are among the foods sold in such places and mostly preferred by low-income consumers. Due to poorly managed production processes, poor harvesting conditions, long drying times in the open air, and poor storage practices, grains are subject to a wide variety of fungal contamination from farm to fork [3].

Production of mycotoxins in cereals is largely dependent on factors such as pre- and/or post-harvest temperature, moisture content, and insect damage. Climate is the main determinant of fungal formation and mycotoxin production [4,5,6]. Some of the mycotoxins are subdivided into field mycotoxins, which are mainly produced by Fusarium before or just after harvest in the crop, and the other part as storage mycotoxins secreted by Aspergillus and Penicillium species during the drying of grains, conversion into commercial products, storage, and sale [4,6]. Mycotoxins are heat-resistant natural toxic compounds with a high bioaccumulation ability [7]. The fact that mycotoxins are resistant to cooking methods paves the way for serious health problems [8]. For this reason, because they may contain mycotoxins, controlling these foods is of great importance in terms of food safety and protection of consumer health.

Many countries have made important regulations on “acceptable health risks” to control mycotoxin contamination in foods and prohibit the trade of contaminated products. These regulations generally depend on a country’s level of economic development, the rate of consumption of high-risk products, and the susceptibility of crops to contamination [9,10]. There is a Turkish Food Codex Contaminants Regulation in force for these products in Türkiye (Table 1). Cereal-based foods are often contaminated with a large number of mycotoxins, but legal regulations have not been prepared considering the health risks associated with the co-existence of mycotoxins. The interactions of mycotoxins in foods can be classified into three main categories: antagonistic, additive, and synergistic. In particular, the additive and synergistic effect may cause the picture to become more intractable in terms of public health [4].

Most of the studies in the literature [11,12,13,14,15] have focused on the presence of a single mycotoxin and the risks it poses. In recent years, various studies on AFs, OTA, ZEN, FUM, and Trichothecenes (TCTs) reveal that mycotoxins naturally coexist in a variety of foods [4].

**Table 1:** Turkish Food Codex contaminant limits of cereal and cereal-based products [16].

Mycotoxins	Food Type			
	Cornmeal	Extruded Maize Snack	Maize-Based Breakfast Cereal	Oatmeal
TAF (μg·kg^−1^)	4	4	4	4
AFB_1_ (μg·kg^−1^)	2	2	2	2
OTA (μg·kg^−1^)	3	3	3	3
DON (μg·kg^−1^)	750	500	500	750
ZEN (μg·kg^−1^)	200	50	50	75
FUM (μg·kg^−1^)	1000	400	400	-

TAF: Total aflatoxin; AFB_1_: Aflatoxin B_1_; DON: Deoxynivalenol; OTA: Ochratoxin A; ZEN: Zearalenone; FUM: Total Fumonisin.

This study aimed to determine the presence, amount, and co-occurrence of multi-mycotoxins (aflatoxin B_1_, B_2_, G_1_, G_2_, ochratoxin, zearalenone (ZEN), deoxynivalenol (DON), fumonisin B_1_ (FB_1_), fumonisin B_2_ (FB_2_), citrinin (CIT), and HT-2 and T-2 toxins) in cereal-based foods with an economical, fast, and reliable LC-MS/MS technique.

## 2. Materials and Methods

### 2.1. Sample Collection

In this study, 140 cereal-based (cornmeal (n:41), extruded maize snacks (n:32), maize-based breakfast cereals (n:27), and oatmeal (n:40)) samples were purchased from temporary open-air markets in suburbs of İstanbul. Samples were collected in nylon bags (100 g) from May 2022 to August 2022. The samples were delivered to the laboratory in their original packages and were stored at 7 °C until analysis.

As part of our research, we took a closer look at the techniques utilized by Kim et al. [17], De Santis et al. [18], and Solfrizzo et al. [19], and made some modifications to them for further examination.

### 2.2. Preparation of Mobile Phases

The extraction solution was prepared by mixing 800 mL of methanol (Merck, Germany) with 200 mL of LC-MS-grade water (Merck, Darmstadt, Germany). The mobile phases were constituted of 0.252 g of ammonium formate (Merck, Darmstadt, Germany) in 1 L of LC-MS-grade water containing 1 mL formic acid (mobile phase A) and 0.252 g of ammonium formate in 1 L of LC-MS-grade methanol containing 1 mL of formic acid (Merck, Darmstadt, Germany) (mobile phase B).

### 2.3. Sample Preparation and Extraction

The samples were homogenised in the grinder (Retsch GM 200, Haan, Germany). Five grams of the samples were weighed. The weighed samples were mixed with 12 mL of the extraction solution for two hours while shaken. At the end of the period, the samples were centrifuged at 3000× *g* for 5 min, passed through a 0.45 µm filter (Millipore, Merck KgaA, Darmstadt, Germany) and collected in a 15 mL centrifuge tube. The filtrate was transferred into a 1.5 mL vial, with a volume of 800 µL. After adding 200 µL of mobile phase A, the solution was analysed. Two replicates were performed in one day.

### 2.4. LC-MS/MS Equipment

LC-MS/MS analysis was performed using an Agilent 6420 Triple Quad/G6420A LC-MS system. Chromatographic separation was performed on an Athena C18-WP column (CNW Technologies GmbH, Düsseldorf, Germany) (100, 50 mm × 2.1 mm × 3 µm particle size) at a column temperature of 35 °C. A gradient program was set up as follows: 0–3 min with 75% A, 25% B, 3.0–5.0 min linear gradient down to 30% A, 70% B; hold at 0% A for 4 min; return to 100% A in 0.1 min (total run time 7.1 min). The flow rate was 0.35 mL·min^−1^ and the injection volume was 20 µL. The LC flow was directed into the MS detector for between 1.0 and 7.0 min. Delta EMV for MS analysis: 400(+), 400(−); Gas Temperature: 350 °C; Gas Flow: 11 L·min^−1^, Nebulizer: 30 psi; and Capillary: 3500 V (+), 2500 V (−). Optimised parameters are summarised in Table S1.

### 2.5. Recovery Rate

Mycotoxin standards of AFB_1_, AFB_2_, AFG_1_, AFG_2_, OTA, FB_1_, and FB_2_ were purchased from the Pa group (Ankara, Türkiye); DON, ZEN, CIT, T-2, and HT-2 were from Trilogy (Washington, DC, USA) in liquid form 1000 mg·L^−1^ and they were stored at 4 °C. The standard solutions for AFB_1_, AFB_2_, AFG_1_, AFG_2_, and OTA were diluted 0.1–1–2.5–5–10 mg·L^−1^; for ZEN, HT-2 toxin, T-2 toxin; for CIT 1–5–10–25–50–100 mg·L^−1^; for FB_2_ 25–50–125–250–500 mg·L^−1^; and for DON and FB_1_ 50–100–250–500–1000 mg·L^−1^. The LC-MS/MS method was validated to investigate performance characteristics such as linearity, the limit of detection (LOD), the limit of quantification (LOQ), and accuracy, according to the European Commission [20]. Linear regression analysis was performed for a standard mycotoxin mixture of AFB_1_, AFB_2_, AFG_1_, AFG_2_, FB_1_, FB_2_, T-2, HT-2, OTA, DON, and ZEN under optimised LC-MS/MS conditions. External calibration was performed. To ensure the peak measurements’ accuracy, we ensured the signal area was at least three times larger than the noise area. During the LOD and LOQ studies, oatmeal, wheat flour, maize flour, and breakfast cereals containing oats were used. We conducted a blank sample analysis to verify that no mycotoxins were present in the samples. Upon observation, 100 μg·kg^−1^ for DON; 0.3 μg·kg^−1^ for AFB_1_ and AFB_2_; 0.4 μg·kg^−1^ for AFG_1_ and AFG_2_; 0.8 μg·kg^−1^ for OTA; 25 μg·kg^−1^ for FB1; 60 μg·kg^−1^ for FB2; 100 μg·kg^−1^ for CIT; and 10 μg·kg^−1^ for ZEN, T-2, and HT-2 spiking levels were performed. Spiking was performed to determine LOD and LOQ. This study was conducted by two different technicians (to also put forth the analysis competencies). Ten measurements with two replicates were taken for each mycotoxin in matrices. The standard deviation (SD) of the measurement data, LOD, and LOQ were calculated using specific Formulas (1)–(3).

(1) $$\mathrm{where}:\;SD=\sqrt{\frac{1}{n}}\sum_{i=1}^{n}{\left(xi-xmean\right)}^{2}$$

(2) where: LOD=SD×3

(3) where: LOQ=SD×10

*LOD*: limit of detection;
*LOQ*: limit of quantification;
*SD*: standard deviation;
*xmean*: the arithmetic mean of the data;
*xi*: each value of the dataset;
*n*: the total number of data.

For the determination of measurement uncertainties, ISO [21] and Ellison and Williams [22] references were used. Repeatability, reproducibility, and uncertainty budgets from recovery were combined as indicated in the references. Uncertainty budgets for each active ingredient were calculated separately. Uncertainties about the equipment used were included in the repeatability, reproducibility, and recovery uncertainty budgets. LOD, LOQ, recovery, and standard uncertainty ratios are given in Table S2, R2 and regression equations are provided in Table S3.

## 3. Results

The results for our cereal-based products are presented in Table 2. We conducted 12 mycotoxin analyses on the collected samples using the LC-MS/MS technique. The findings revealed that 28.57% of the samples exceeded the maximum limits specified in the Turkish Food Codex Contaminants Regulation in terms of AFB_1_ and 26.43% in terms of total aflatoxin.

It was determined that 74.29% of the samples were contaminated with AFB_1_, 45% with AFB_2_, 36.43% with AFG_1_, and 40.71% with AFG_2_ at levels above the detectable limits. While this rate was 22.86% for FB_1_, contamination with FB_2_ was detected in 90% of samples. On the other hand, 87.86% of the samples were contaminated with OTA, 44.29% with T-2, 40.71% with HT-2, and 23.57% with ZEN and DON. CIT was not detected in any of the samples.

The mean concentration of the samples with AFB_1_ was calculated as 2.01 ± 1.6 µg·kg^−1^, and the median was determined as 1.65 µg·kg^−1^. The mean AFB_2_, AFG_1_, and AFG_2_ values of the positive samples were calculated as 2.39 ± 0.18 µg·kg^−1^, 2.41 ± 0.07 µg·kg^−1^, and 2.29 ± 1.84 µg·kg^−1^, respectively. The positive samples’ mean TAF concentration was 3.79 ± 4.14 μg·kg^−1^.

The mean concentration of the samples with FB_1_ was calculated as 146.17 ± 7.75 µg·kg^−1^, and the median was determined as 142.46 µg·kg^−1^. The mean FB_2_ concentration was 97.97 ± 58.02 µg·kg^−1^ and the median was 59.99 µg·kg^−1^.

OTA, DON, ZEN, and HT-2 values of the positive samples were calculated as 1.3 ± 0.002 µg·kg^−1^, 171.26 ± 2.63 µg·kg^−1^, 16.41 ± 0.34 µg·kg^−1^, and 35.17 ± 8.65 µg·kg^−1^, respectively. None of the samples’ T-2 values were above the LOQ. The positive samples’ median concentration of that were 1.30 μg·kg^−1^, 170.64 μg·kg^−1^, 16.32 μg·kg^−1^, and 38.91 μg·kg^−1^.

## 4. Discussion

In our study, multiple mycotoxin analyses in cereal-based foods (n:140) were performed using the liquid chromatography–tandem mass chromatography method, which provides a fast, reliable, and economic analysis (n:140). LC-MS/MS is mostly preferred for mycotoxin analysis due to its high selectivity and sensitivity. According to a report by Amirahmadi et al. [23], the matrix effect could potentially have a detrimental effect on quantitative performance, particularly as the electrospray ionization (ESI) source is approached. The methods used in our study meet the requirements of EC No: 401/2006 [24] and EC No: 1881/2006 [25] commission regulations. The data we reported on mycotoxin contamination in cereal-based products in Türkiye provide crucial data in terms of the diversity of analytes investigated, including some less frequently investigated secondary fungal metabolites, which were first reported in products offered for sale in open-market places.

The EU stated that no direct human consumption product should be present at levels higher than 2 µg·kg^−1^ for AFB_1_ and more than 4 µg·kg^−1^ for total aflatoxin [25,26]. The regulation in TFC is also in this direction for grain-based products that make up our sample set [16].

In our study, it was observed that none of the cornmeal samples exceeded the limit value reported in TFC for AFB_1_ and TAF. On the other hand, 95% and 90% of oatmeal samples were found to be above the limit values for AFB_1_ and TAF, respectively. The sample rate exceeding the AFB_1_ and TAF limit values in extruded maize snack products was determined as 3.13%. However, 3.7% of maize-based breakfast cereal products exceeded the AFB1 limit while none of the samples exceeded the TAF limit value.

There are many studies in the literature about the presence of aflatoxins in corn and oat-based samples. Alborch et al. [27] found that the samples were contaminated with 0.19–3.1 μg·kg^−1^ of aflatoxin in their study on 60 cornmeal and corn grain samples. Similarly, Zhou et al. [28] also detected AFB_2_ in the range of 0.67–1.3 μg·kg^−1^ in 16 corn samples they examined. In another study, the mean AFB_2_ level was measured in 79 different types of corn samples, with very little difference, but still close (1.5 μg·kg^−1^) [29]. When the data in the mentioned studies were compared with those in our study, it was noticed that they were similar. However, in some studies, it was found that the average aflatoxin values in the samples examined were higher than our data [17,30,31,32], while in some others, they were below the detection limits, in other words, at very low levels contrary to ours [33,34,35].

In terms of oat-based products, there is a slight difference in the presence of aflatoxin compared to corn-based products. For example, in some studies, it is reported that the types of aflatoxins detected in oat-based products are more limited, but also their levels are lower. According to Ortiz et al. [36]’s study, only 2% of the 42 oat flakes samples they examined were contaminated with AFB_1_, while other aflatoxin types could not be detected. Juan et al. [37] also stated that AFG_1_ was found in only 0.1% of the samples they analysed, while other types were not present. This has been associated with the correct functioning of the food safety system in the processing of products. In addition, in some studies, it has been reported that the total amount of aflatoxins in the oat-based samples examined was below the detection limits, in other words, no type of aflatoxins was found in these products [38,39,40,41]. It is predicted by the researchers that this situation may be caused by various factors such as product type differences, processing techniques, and temperature changes encountered in the process. However, there are also studies reporting a higher rate of aflatoxin content in oat products. For example, Al-Taher et al. [42] reported that they found AFB_2_ at the level of 1.1–2.6 μg·kg^−1^ and AFG_2_ at the level of 0.4–1.7 μg·kg^−1^ in 40% of the 20 breakfast samples they examined. Additionally, Kuzdralinski et al. [43] stated that 41% of 58 breakfast oat samples they analysed were contaminated with aflatoxin at an average level of 1.4 μg·kg^−1^.

Fumonisin, one of the most toxic types of mycotoxins known, unlike aflatoxins, multiplies by contaminating the developing crop prior to harvest, so that, generally, it is difficult to control or prevent its development. There are three types of fumonisin, FB_1_, FB_2_, and FB_3_, and FB_1_ is the most common type in foods, especially in corn and corn-based products. The fact that fumonisin B_1_ (average 146.17 μg·kg^−1^) was detected only in corn-based samples among the samples examined within the framework of our study is a finding that confirms this information. In the literature review, it is seen that the studies mostly focus on FB_1_. However, although it is not as strong as FB_1_ regarding toxicity potential, it is also known that if FB_2_ is present in the flora, it increases toxicity by showing a synergistic effect with FB_1_. Among the samples in this study, an average of 58–60 μg·kg^−1^ was detected in corn-based samples and an average of 166.55 μg·kg^−1^ in oat-based samples. Many studies in the literature also support our findings from corn products [17,33,44]. On the other hand, there are also studies reporting that fumonisin species are less common in oat-based products compared to corn-based products [29,42,45].

In addition to the toxins we mentioned in corn and oat-based agricultural products, other toxins can be found. For example, heterogeneous trichothecenes and mycooestrogens (type A: T-2 and HT-2; type B: DON, ZEN, and CIT) produced by some Fusarium species fungi and different Aspergillus species (*A. ochraceus*, *A. steyni*, *A. westerdijkiae*) and OTA, classified in Group 2, are the most known toxins for humans in this regard. The presence of these toxins, as with the toxins we mentioned before, is mostly affected by errors in agricultural practices, storage defects, insect attacks, genetic factors, and negative climatic conditions.

According to the data of our study, it was observed that those toxins instead of OTA were found at higher rates and values in oat-based products compared to corn-based ones, while OTA was found to be higher only in corn-based products (Table 2). Moreover, in the results section we mentioned, T-2 remained below the detection limits in the products we examined, while CIT could not be detected in any of the samples. In this context, it is known that there are studies on the presence of multi-mycotoxin equivalent to our study. Alborch et al. [27] detected OTA at a level similar to our study (0.79–1.71 μg·kg^−1^) in 33.3% of 60 corn-based products they collected from open markets, while Kim et al. [17] reported that they detected 180.4 μg·kg^−1^ DON in 13% of 507 corn samples in their study. In another study, researchers reported that corn samples (n:37) were contaminated with DON (100%; 171 μg·kg^−1^) and OTA (19%; 1 μg·kg^−1^) equivalent to our data [46].

In a study on oat-based products, it was reported that approximately 50% of the 20 oat breakfast samples were contaminated with DON, OTA, and ZEN at levels of 2.5–146.5 μg·kg^−1^, 0.6–11.8 μg·kg^−1^, and 1.2–13.6 μg·kg^−1^, respectively [42]. There are also studies in which the mycotoxin levels detected are much lower [34,37,39,44] and much higher [40,43,47,48,49] when compared to the data of our study.

**Figure 1:**
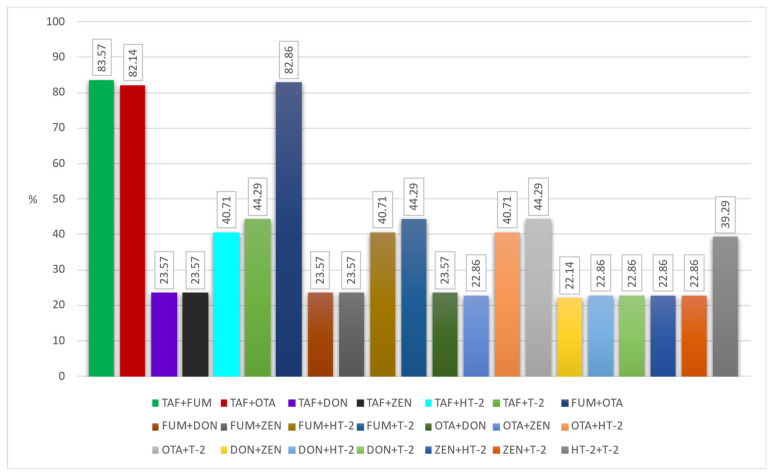
The co-occurrence of two mycotoxin types.

**Figure 2:**
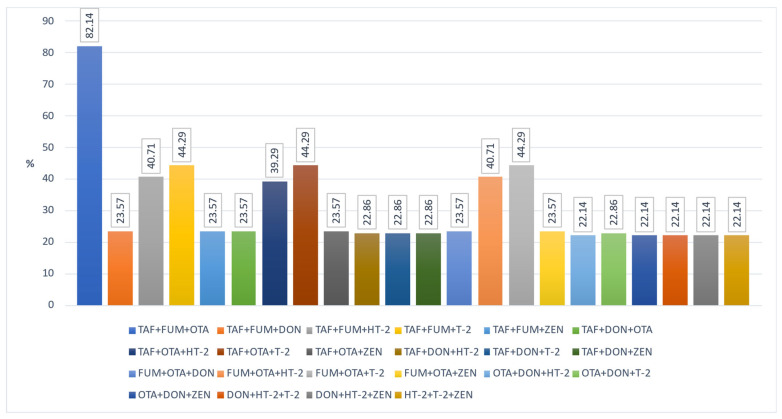
The co-occurrence of three mycotoxin types.

**Figure 3:**
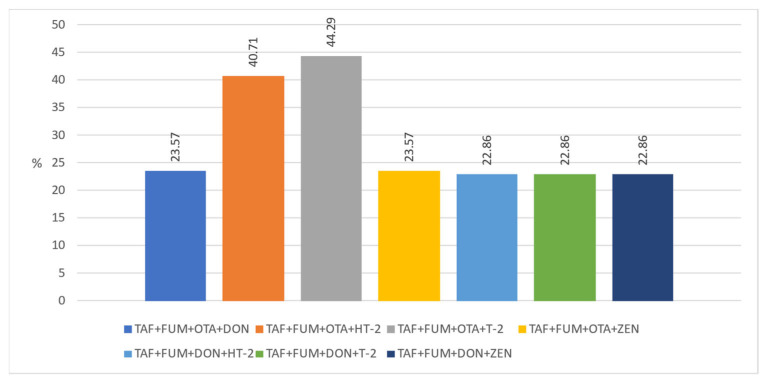
The co-occurrence of four mycotoxin types.

**Figure 4:**
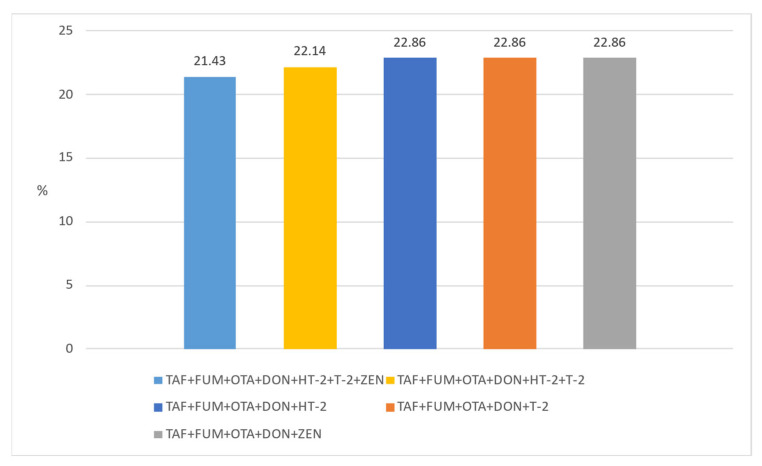
The co-occurrence of five and more mycotoxin types.

Another important issue that needs to be emphasized is that these toxins can be found in different and multiple combinations in foods (natural co-occurrence). This can cause toxins to interact with each other in undesirable ways. TAF and FUM, DON and ZEN, TAF and OTA, and FUM and ZEN are mostly observed in the literature in cereals and grain product samples. However, due to the synergistic negative effects of co-occurring mycotoxins on human and animal health, it is important to evaluate their co-occurrence [50]. Unfortunately, it seems that most studies have focused on the formation of a single mycotoxin. Only a few studies reported the number of co-occurring mycotoxins and the proportion of co-contaminated samples together, as well as the main combinations found [4]. Moreover, legal regulations do not consider the combined effects of mycotoxins. In this context, we evaluated the natural co-occurrence of these toxins in our samples. In our study, the co-occurrence rates of any two mycotoxins in the samples are given in Figure 1. The co-existence of any three mycotoxins in our samples is indicated in Figure 2. ZEN + DON + OTA was found in 22.14% of the samples, DON + TAF + FUM in 23.57%, and TAF + ZEN + FUM in 23.57%. In addition, in Figure 3 and Figure 4, the co-existence rates of four or more mycotoxins in our samples are shown.

## 5. Conclusions

This research contributes to eliminating the deficiency observed in the literature regarding the presence and concentration of mycotoxins in cereal-based products marketed in open-air markets in Türkiye. Mycotoxin contaminations, mycotoxin combinations, contamination status, and frequency of occurrence in these foodstuffs were determined. It has been revealed that effective control of mycotoxins, which may pose a risk to human health even at low doses, should be carried out with monitoring systems in this and similar types of foodstuffs. On the other hand, considering the additive and synergistic interactions of different mycotoxins, new legal regulations will be needed regarding the presence and limit values of mycotoxin combinations in foods. It is highly recommended to continuously monitor the mycotoxins that were studied in vulnerable food items and to conduct research aimed at identifying masked mycotoxins and other fungal metabolites. Additionally, efforts should be focused on reducing and managing mycotoxins within the food supply chain.

## Figures and Tables

**Table 2:** Descriptive statistical data of all cereal based samples (n:140) *.

AFB_1_	LOD > (<0.24 μg·kg^−1^) (n)	LOD-LOQ > (0.24–0.78 > μg·kg^−1^) (n)	≥0.78–1 μg·kg^−1^ (n)	≥1–2 μg·kg^−1^ (n)	≥2 μg·kg^−1^ (n)	*x* μg·kg^−1^	SD μg·kg^−1^	Med μg·kg^−1^
Cornmeal	34	4	1	2	-	1.01	0.07	1.01
Extruded Maize Snack	2	2	8	19	1	1.30	0.46	1.20
Maize Based Breakfast Cereal	-	-	2	24	1	1.39	0.35	1.33
Oatmeal	-	-	-	2	38	3.00	2.11	2.55
TOTAL	36	6	11	47	40	2.01	1.60	1.65
**AFB_2_**	**LOD>** **(<0.26 μg·kg^−1^)** **(n)**	**LOD-LOQ >** **(0.26–0.82 > μg·kg^−1^)** **(n)**	**≥0.82–1 μg·kg^−1^** **(n)**	**≥1–2 μg·kg^−1^** **(n)**	**≥2 μg·kg^−1^** **(n)**	***x*** **μg·kg^−1^**	**SD** **μg·kg^−1^**	**Med** **μg·kg^−1^**
Cornmeal	19	22	-	-	-	-	-	-
Extruded Maize Snack	25	7	-	-	-	-	-	-
Maize Based Breakfast Cereal	27	-	-	-	-	-	-	-
Oatmeal	6	-	-	-	34	2.39	0.18	2.35
TOTAL	77	29	-	-	34	2.39	0.18	2.35
**AFG_1_**	**LOD>** **(<0.22 μg·kg^−1^)** **(n)**	**LOD-LOQ >** **(0.22–0.74 > μg·kg^−1^)** **(n)**	**≥0.74–1 μg·kg^−1^** **(n)**	**≥1–2 μg·kg^−1^** **(n)**	**≥2 μg·kg^−1^** **(n)**	***x*** **μg·kg^−1^**	**SD** **μg·kg^−1^**	**Med** **μg·kg^−1^**
Cornmeal	30	11	-	-	-	-	-	-
Extruded Maize Snack	25	7	-	-	-	-	-	-
Maize Based Breakfast Cereal	27	-	-	-	-	-	-	-
Oatmeal		-	-	-	33	2.41	0.07	2.38
TOTAL	89	18	-	-	33	2.41	0.07	2.38
**AFG_2_**	**LOD>** **(<0.24 μg·kg^−1^)** **(n)**	**LOD-LOQ >** **(0.24–0.78 > μg·kg^−1^)** **(n)**	**≥0.78–1 μg·kg^−1^** **(n)**	**≥1–2 μg·kg^−1^** **(n)**	**≥2 μg·kg^−1^** **(n)**	***x*** **μg·kg^−1^**	**SD** **μg·kg^−1^**	**Med** **μg·kg^−1^**
Cornmeal	21	6	4	7	3	1.58	0.74	1.43
Extruded Maize Snack	25	2	-	-	5	4.27	4.77	2.04
Maize Based Breakfast Cereal	27	-	-	-	-	-	-	-
Oatmeal	10	-	-	17	13	2.29	1.15	1.89
TOTAL	83	8	4	24	21	2.29	1.84	1.82
**TAF**	**LOD>** **(<0.22 μg·kg^−1^)** **(n)**	**LOD-LOQ>** **(0.22–0.74 > μg·kg^−1^)** **(n)**	**≥0.74–2 μg·kg^−1^** **(n)**	**≥2–4 μg·kg^−1^** **(n)**	**≥4 μg·kg^−1^** **(n)**	***x*** **μg·kg^−1^**	**SD** **μg·kg^−1^**	**Med** **μg·kg^−1^**
Cornmeal	15	9	14	3	-	1.58	0.74	0.43
Extruded Maize Snack	-	-	24	7	1	1.93	2.16	1.31
Maize Based Breakfast Cereal	-	-	26	1	-	1.39	0.35	1.33
Oatmeal	-	-	2	2	36	8.74	3.56	8.99
TOTAL	15	9	66	13	37	3.79	4.14	1.65
**FB_1_**	**LOD>** **(<31.14 μg·kg^−1^)** **(n)**	**LOD-LOQ >** **(31.14–103.79 > μg·kg^−1^)** **(n)**	**≥103.79–150 μg·kg^−1^** **(n)**	**≥150–200 μg·kg^−1^** **(n)**	**≥200 μg·kg^−1^** **(n)**	***x*** **μg·kg^−1^**	**SD** **μg·kg^−1^**	**Med** **μg·kg^−1^**
Cornmeal	18	21	2	-	-	141.98	0.0006	141.98
Extruded Maize Snack	25	5	1	1		150.36	10.49	150.36
Maize Based Breakfast Cereal	27	-	-	-	-	-	-	-
Oatmeal	38	2	-	-	-	-	-	-
TOTAL	108	28	3	1	-	146.17	7.75	142.46
**FB_2_**	**LOD>** **(<17.11 μg·kg^−1^)** **(n)**	**LOD-LOQ>** **(17.11–57.04 > μg·kg^−1^)**	**≥57.04–100 μg·kg^−1^** **(n)**	**≥100–200 μg·kg^−1^** **(n)**	**≥200 μg·kg^−1^** **(n)**	***x*** **μg·kg^−1^**	**SD** **μg·kg^−1^**	**Med** **μg·kg^−1^**
Cornmeal	14	12	15	-	-	58.38	0.95	58.18
Extruded Maize Snack	-	2	30	-	-	60.24	4.28	59.07
Maize Based Breakfast Cereal	-	-	27	-	-	60.34	3.15	59.07
Oatmeal	-	-	6	34	-	166.55	45.36	182.93
TOTAL	14	14	78	34	-	97.97	58.02	59.99
**FUM**	**LOD>** **(<17.11 μg·kg^−1^)** **(n)**	**LOD-LOQ>** **(17.11–57.04 > μg·kg^−1^)** **(n)**	**≥57.04–200 μg·kg^−1^** **(n)**	**≥200–800 μg·kg^−1^** **(n)**	**≥800 μg·kg^−1^** **(n)**	***x*** **μg·kg^−1^**	**SD** **μg·kg^−1^**	**Med** **μg·kg^−1^**
Cornmeal	14	12	13	2	-	82.05	56.04	58.18
Extruded Maize Snack	-	2	28	2	-	65.87	41.57	58.92
Maize Based Breakfast Cereal	-	-	27	-	-	60.34	3.15	59.07
Oatmeal	-	-	40	-	-	166.55	45.36	182.39
TOTAL	14	14	108	4	-	106.31	62.25	59.94
**OTA**	**LOD>** **(<0.26 μg·kg^−1^)** **(n)**	**LOD-LOQ>** **(0.26–0.85 > μg·kg^−1^)** **(n)**	**≥0.85–1 μg·kg^−1^** **(n)**	**≥1–3 μg·kg^−1^** **(n)**	**≥3 μg·kg^−1^** **(n)**	***x*** **μg·kg^−1^**	**SD** **μg·kg^−1^**	**Med** **μg·kg^−1^**
Cornmeal	14	27	-	-	-	-	-	-
Extruded Maize Snack	-	32	-	-	-	-	-	-
Maize Based Breakfast Cereal	3	24	-	-	-	-	-	-
Oatmeal	-	6	-	34	-	1.30	0.002	1.30
TOTAL	17	89	-	34	-	1.30	0.002	1.30
**DON**	**LOD>** **(<28.51 μg·kg^−1^)** **(n)**	**LOD-LOQ >** **(28.51–95.03 > μg·kg^−1^)** **(n)**	**≥95.03–150 μg·kg^−1^** **(n)**	**≥150–500 μg·kg^−1^** **(n)**	**≥500 μg·kg^−1^** **(n)**	***x*** **μg·kg^−1^**	**SD** **μg·kg^−1^**	**Med** **μg·kg^−1^**
Cornmeal	41	-	-	-	-	-	-	-
Extruded Maize Snack	32	-	-	-	-	-	-	-
Maize Based Breakfast Cereal	26	1	-	-	-	-	-	-
Oatmeal	7	-	-	33	-	171.26	2.63	170.64
TOTAL	106	1	-	33	-	171.26	2.63	170.64
**ZEN**	**LOD >** **(<2.94 μg·kg^−1^)** **(n)**	**LOD-LOQ >** **(2.94–9.80 > μg·kg^−1^)** **(n)**	**≥9.80–50 μg·kg^−1^** **(n)**	**≥50–100 μg·kg^−1^** **(n)**	**≥100 μg·kg^−1^** **(n)**	***x*** **μg·kg^−1^**	**SD** **μg·kg^−1^**	**Med** **μg·kg^−1^**
Cornmeal	41	-	-	-	-	-	-	-
Extruded Maize Snack	32	-	-	-	-	-	-	-
Maize Based Breakfast Cereal	27	-	-	-	-	-	-	-
Oatmeal	7	-	33	-	-	16.41	0.34	16.32
TOTAL	107	-	33	-	-	16.41	0.34	16.32
**HT-2**	**LOD >** **(<3.32 μg·kg^−1^)** **(n)**	**LOD-LOQ >** **(3.32–11.08 > μg·kg^−1^)** **(n)**	**≥11.08–20 μg·kg^−1^** **(n)**	**≥20–40 μg·kg^−1^** **(n)**	**≥40–50 μg·kg^−1^** **(n)**	***x*** **μg·kg^−1^**	**SD** **μg·kg^−1^**	**Med** **μg·kg^−1^**
Cornmeal	22	13	5	1	-	-	-	-
Extruded Maize Snack	27	3	2		-	16.07	1.63	16.07
Maize Based Breakfast Cereal	27	-	-	-	-	-	-	-
Oatmeal	7	-	-	27	6	39.29	1.36	39.09
TOTAL	83	16	7	28	6	35.17	8.65	38.91
**T-2**	**LOD >** **(<3.16 μg·kg^−1^)** **(n)**	**LOD-LOQ >** **(3.16–10.55 > μg·kg^−1^)** **(n)**	**≥10.55–50 μg·kg^−1^** **(n)**	**≥50–100 μg·kg^−1^** **(n)**	**≥100 μg·kg^−1^** **(n)**	***x*** **μg·kg^−1^**	**SD** **μg·kg^−1^**	**Med** **μg·kg^−1^**
Cornmeal	19	22	-	-	-	-	-	-
Extruded Maize Snack	25	7	-	-	-	-	-	-
Maize Based Breakfast Cereal	27	-	-	-	-	-	-	-
Oatmeal	7	33	-	-	-	-	-	-
TOTAL	78	62	-	-	-	-	-	-

FB_1_: Fumonisin B_1_; FB_2_: Fumonisin B_2_; HT-2: HT-2 toxin; OTA: Ochratoxin A; TAF: Total Aflatoxin; FUM: Total Fumonisin; T-2: T-2 toxin; ZEN: Zearalenone; LOQ: Limit of quantification; LOD: Limit of detection; SD: standard deviation; x: mean; Med: median. * When calculating x, SD, and the median, only values above the LOQ are included.

## Data Availability

The data used to support the findings of this study are available by the corresponding author upon request.

## Supplementary Materials

The following supporting information can be downloaded at: https://www.mdpi.com/article/10.3390/foods12142744/s1, Table S1: MS-MS ion transitions, Table S2: Recovery results in multitoxin analysis, Table S3: R2 and regression equation.

## References

[1] Clark W.A.V., Thrall G.I. (2020). Human Migration.

[2] Sik E., Wallace C. (1999). The development of open-air markets in East-Central Europe. Int. J. Urban Reg. Res..

[3] Yogendrarajah P., Jacxsens L., De Saeger S., De Meulenaer B. (2014). Co-occurrence of multiple mycotoxins in dry chilli (*Capsicum annum* L.) samples from the markets of Sri Lanka and Belgium. Food Control.

[4] Smith M.-C., Madec S., Coton E., Hymery N. (2016). Natural Co-Occurrence of Mycotoxins in Foods and Feeds and Their in vitro Combined Toxicological Effects. Toxins.

[5] Manu N., Opit G.P., Osekre E.A., Arthur F.H., Mbata G., Armstrong P., Danso J.K., McNeill S.G., Campbell J.F. (2019). Moisture content, insect pest infestation and mycotoxin levels of maize in markets in the northern region of Ghana. J. Stored Prod. Res..

[6] Saeed F., Afzaal M., Niaz B., Rasheed A., Umar M., Hussain M., Ahmad Nayik G., Ansari M.J., Nayik G.A., Tufail T., Anjum F.M., Ansari M.J. (2023). Quality and Safety Aspects of Cereal Grains. Cereal Grains: Composition, Nutritional Attributes, and Potential Applications.

[7] Majeed S., De Boevre M., De Saeger S., Rauf W., Tawab A., Fazal-e-Habib, Rahman M., Iqbal M. (2018). Multiple Mycotoxins in Rice: Occurrence and Health Risk Assessment in Children and Adults of Punjab, Pakistan. Toxins.

[8] Palumbo R., Crisci A., Venâncio A., Cortiñas Abrahantes J., Dorne J.-L., Battilani P., Toscano P. (2020). Occurrence and Co-Occurrence of Mycotoxins in Cereal-Based Feed and Food. Microorganisms.

[9] Kendra D.F., Dyer R.B. (2007). Opportunities for biotechnology and policy regarding mycotoxin issues in international trade. Int. J. Food Microbiol..

[10] Udomkun P., Wiredu A.N., Nagle M., Müller J., Vanlauwe B., Bandyopadhyay R. (2017). Innovative technologies to manage aflatoxins in foods and feeds and the profitability of application–A review. Food Control.

[11] Jindal N., Mahipal S.K., Rottinghaus G.E. (1999). Occurrence of fumonisin B1 in maize and poultry feeds in Haryana, India. Mycopathologia.

[12] Belli N., Marin S., Sanchis V., Ramos A.J. (2002). Ochratoxin A (OTA) in wines, musts and grape juices: Occurrence, regulations and methods of analysis. FSTI.

[13] Scudamore K.A., Patel S. (2009). Occurrence of Fusarium mycotoxins in maize imported into the UK, 2004–2007. Food Addit. Contam. Part A Chem. Anal. Control Expo. Risk Assess..

[14] Scussel V.M., Savi G.D., Costas L.L.F., Xavier J.J.M., Manfio D., Bittencourt K.O., Agular K., Stein S.M. (2014). Fumonisins in corn (*Zea mays* L.) from Southern Brazil. Food Addit. Contam. Part B Surveill..

[15] Burlakoti R.R., Tamburic-Ilincic L., Limay-Rios V., Burlakoti P. (2017). Comparative population structure and trichothecene mycotoxin profiling of *Fusarium graminearum* from corn and wheat in Ontario, central Canada. Plant Pathol..

[16] Turkish Food Codex Regulation (TFC) (2011). Communiqué on Determination of Maximum Levels of Certain Contaminants in Foodstuffs. Off. Gaz..

[17] Kim D.-H., Hong S.-Y., Kang J.W., Cho S.M., Lee K.R., An T.K., Lee C., Chung S.H. (2017). Simultaneous Determination of Multi-Mycotoxins in Cereal Grains Collected from South Korea by LC/MS/MS. Toxins.

[18] De Santis B., Debegnach F., Gregori E., Russo S., Marchegiani F., Moracci G., Brera C. (2017). Development of a LC-MS/MS Method for the Multi-Mycotoxin Determination in Composite Cereal-Based Samples. Toxins.

[19] Solfrizzo M., Gambacorta L., Bibi R., Ciriaci M., Paoloni A., Pecorelli I. (2018). Multimycotoxin analysis by LC-MS/MS in cereal food and feed: Comparison of different approaches for extraction, purification, and calibration. J. AOAC Int..

[20] (2002). EC No 657/2002 Commission Decision of 12 August 2002 Implementing Council Directive 96/23/EC Concerning the Performance of Analytical Methods and the Interpretation of Results (Text with EEA Relevance) (Notified under Document Number C (2002) 3044). Off. J. Eur. Union L.

[21] (2010). Guidance for the Use of Repeatability, Reproducibility and Trueness Estimates in Measurement Uncertainty Estimation.

[22] Ellison S.L., Williams A. (2012). Quantifying Uncertainty in Analytical Measurement.

[23] Amirahmadi M., Shoeibi S., Rastegar H., Elmi M., Mousavi Khaneghah A. (2018). Simultaneous analysis of mycotoxins in corn flour using LC/MS-MS combined with a modified QuEChERS procedure. Toxin Rev..

[24] (2006). EC No 401/2006 of 23 February 2006 Laying Down the Methods of Sampling and Analysis for the Official Control of the Levels of Mycotoxins in Foodstuffs (Text with EEA Relevance). Off. J. Eur. Union L.

[25] (2006). European Commission Regulation (EC) No 1881/2006 of 19 December 2006 Setting Maximum Levels for Certain Contaminants in Foodstuffs. Off. J. Eur. Union L.

[26] (2010). European Commission EC No 165/2010 of 26 February 2010, Amending Regulation (EC) no 1881/2006 Setting Maximum Levels for Certain Contaminants in Foodstuffs as Regards Aflatoxin. Off. J. Eur. Union L.

[27] Alborch L., Bragulat M.R., Castella G., Abarca M.L., Cabanes F.J. (2012). Mycobiota and mycotoxin contamination of maize flours and popcorn kernels for human consumption commercialized in Spain. Food Microbiol..

[28] Zhou J., Xu J.-J., Huang B.-F., Cai Z.-X., Ren Y.-P. (2017). High-performance liquid chromatographic determination of multi-mycotoxin in cereals and bean foodstuffs using interference-removal solid-phase extraction combined with optimized dispersive liquid–liquid microextraction. J. Sep. Sci..

[29] Manizan A.M., Oplatowska-Stachowiak M., Piro-Metayer I., Campbell K., Koffi-Nevry R., Elliott C., Akaki K.D., Montet D., Brabet C. (2018). Multi-mycotoxin determination in rice, maize and peanut products most consumed in Côte d’Ivoire by UHPLC-MS/MS. Food Control.

[30] Warth B., Parich A., Atehnkeng J., Bandyopadhyay R., Schuhmacher R., Sulyok M., Krska R. (2012). Quantitation of Mycotoxins in Food and Feed from Burkina Faso and Mozambique Using a Modern LC-MS/MS Multitoxin Method. J. Agric. Food Chem..

[31] Li X., Liu B., Wang F., Ma X., Li Z., Guo D., Wang Y., Wan F., Deng L., Zhang S. (2018). Determination of 16 Mycotoxins in Maize by Ultrahigh-Performance Liquid Chromatography–Tandem Mass Spectrometry. Anal. Lett..

[32] Tarazona A., Gómez J.V., Mateo F., Jiménez M., Romera D., Mateo E.M. (2020). Study on mycotoxin contamination of maize kernels in Spain. Food Control.

[33] Ediage E.N., Di Mavungu J.D., Monbaliu S., Peteghem C.V., De Saeger S. (2011). A Validated Multianalyte LC-MS/MS Method for Quantification of 25 Mycotoxins in Cassava Flour, Peanut Cake and Maize Samples. J. Agric. Food Chem..

[34] Soleimany F., Jinap S., Faridah A., Khatib A. (2012). A UPLC–MS/MS for simultaneous determination of aflatoxins, ochratoxin A, zearalenone, DON, fumonisins, T-2 toxin and HT-2 toxin, in cereals. Food Control.

[35] Paschoal F.N., De Azevedo Silva D., De Souza R.V.S., de Oliveira M.S., Pereira D.A.A., de Souza S.V.C. (2017). A Rapid Single-Extraction Method for the Simultaneous Determination of Aflatoxins B1, B2, G1, G2, Fumonisin B1, and Zearalenone in Corn Meal by Ultra Performance Liquid Chromatography Tandem Mass Spectrometry. Food Anal. Methods.

[36] Ortiz J., Camp J.V., Mestdagh F., Donoso S., De Meulenaer B. (2013). Mycotoxin co-occurrence in rice, oat flakes and wheat noodles used as staple foods in Ecuador. Food Addit. Contam. Part A.

[37] Juan C., Mañes J., Juan-García A., Moltó J.C. (2022). Multimycotoxin Analysis in Oat, Rice, Almond and Soy Beverages by Liquid Chromatography-Tandem Mass Spectrometry. Appl. Sci..

[38] Vidal A., Marín S., Ramos A.J., Cano-Sancho G., Sanchis V. (2013). Determination of aflatoxins, deoxynivalenol, ochratoxin A and zearalenone in wheat and oat based bran supplements sold in the Spanish market. Food Chem. Toxicol..

[39] Bryła M., Waśkiewicz A., Podolska G., Szymczyk K., Jędrzejczak R., Damaziak K., Sułek A. (2016). Occurrence of 26 Mycotoxins in the Grain of Cereals Cultivated in Poland. Toxins.

[40] Tittlemier S.A., Blagden R., Chan J., Roscoe M., Pleskach K. (2020). A multi-year survey of mycotoxins and ergosterol in Canadian oats. Mycotoxin Res..

[41] Meyer J.C., Hennies I., Wessels D., Schwarz K. (2021). Survey of mycotoxins in milling oats dedicated for food purposes between 2013 and 2019 by LC–MS/MS. Food Addit. Contam. Part A Chem. Anal. Control Expo. Risk Assess..

[42] Al-Taher F., Cappozzo J., Zweigenbaum J., Lee H.J., Jackson L., Ryu D. (2017). Detection and quantitation of mycotoxins in infant cereals in the U.S. market by LC-MS/MS using a stable isotope dilution assay. Food Control.

[43] Kuzdraliński A., Solarska E., Mazurkiewicz J. (2023). Mycotoxin content of organic and conventional oats from southeastern Poland. Food Control.

[44] Martos P.A., Thompson W., Diaz G.J. (2010). Multiresidue mycotoxin analysis in wheat, barley, oats, rye and maize grain by high performance liquid chromatography-tandem mass spectrometry. World Mycotoxin J..

[45] Oueslati S., Romero-González R., Lasram S., Frenich A.G., Vidal J.L.M. (2012). Multi-mycotoxin determination in cereals and derived products marketed in Tunisia using ultra-high performance liquid chromatography coupled to triple quadrupole mass spectrometry. Food Chem. Toxicol..

[46] Abia W.A., Warth B., Sulyok M., Krska R., Tchana A.N., Njobeh P.B., Dutton M.F., Moundipa P.F. (2013). Determination of multi-mycotoxin occurrence in cereals, nuts and their products in Cameroon by liquid chromatography tandem mass spectrometry (LC-MS/MS). Food Control.

[47] De Boevre M., Di Mavungu J.D., Maene P., Audenaert K., Deforce D., Haesaert G., Eeckhout M., Callebaut A., Berthiller F., Van Peteghem C. (2012). Development and validation of an LC-MS/MS method for the simultaneous determination of deoxynivalenol, zearalenone, T-2-toxin and some masked metabolites in different cereals and cereal-derived food. Food Addit. Contam. Part A Chem. Anal. Control Expo. Risk Assess..

[48] Uhlig S., Eriksen G.S., Hofgaard I.S., Krska R., Beltrán E., Sulyok M. (2013). Faces of a Changing Climate: Semi-Quantitative Multi-Mycotoxin Analysis of Grain Grown in Exceptional Climatic Conditions in Norway. Toxins.

[49] Islam M.N., Tabassum M., Banik M., Daayf F., Fernando W.G.D., Harris L.J., Sura S., Wang X. (2021). Naturally Occurring Fusarium Species and Mycotoxins in Oat Grains from Manitoba, Canada. Toxins.

[50] Streit E., Schatzmayr G., Tassis P., Tzika E., Marin D., Taranu I., Tabuc C., Nicolau A., Aprodu I., Puel O. (2012). Current Situation of Mycotoxin Contamination and Co-occurrence in Animal Feed—Focus on Europe. Toxins.

