# Supplementary material for: Determination of Multimycotoxin in Cereal-Based Products Sold in Open-Air Markets
Source: Foods. 2023 Jul 19;12(14):2744. doi: 10.3390/foods12142744 (PMC10380067; doi:10.3390/foods12142744)
Supplement: Supplementary file 1 [file foods-12-02744-s001.zip › foods-2476954-supplementary.pdf]

Table S1. MS-MS ion transitions

Mycotoxin	Precursor Ion m/z	Product Ion m/z	Ret Time (min)	Delta Ret Time (min)	Fragmentor (V)	Collision Energy (V)	Cell Accelerator Voltage (V)	Polarity
**AFB_1**	313.2	269.9	4.80	1.08	130	28	7	Positive
**AFB_1**	313.2	257.0	4.80	1.08	130	24	7	Positive
**AFB_1**	313.2	285.2	4.80	1.08	130	12	7	Positive
**AFB_2**	315.1	287.0	4.64	1.18	130	24	7	Positive
**AFB_2**	315.1	259.0	4.64	1.18	130	32	7	Positive
**AFB_2**	315.1	243.0	4.64	1.18	130	44	7	Positive
**AFG_1**	329.1	311.0	4.43	1.06	100	20	7	Positive
**AFG_1**	329.1	282.9	4.43	1.06	100	24	7	Positive
**AFG_1**	329.1	243.1	4.43	1.06	100	28	7	Positive
**AFG_2**	331.0	313.0	4.25	0.73	90	24	7	Positive
**AFG_2**	331.0	245.0	4.25	0.73	90	32	7	Positive
**AFG_2**	331.0	216.9	4.25	0.73	90	40	7	Positive
**CIT**	251.1	233.0	5.39	1.13	88	12	7	Positive
**CIT**	251.1	205.0	5.39	1.13	88	28	7	Positive
**CIT**	251.1	91.0	5.39	1.13	88	50	7	Positive
**DON**	297.1	249.1	2.39	2.82	90	8	7	Positive
**DON**	297.1	231.0	2.39	2.82	90	8	7	Positive
**DON**	297.1	202.9	2.39	2.82	90	12	7	Positive
**FB_1**	722.4	704.3	5.11	0.99	116	32	7	Positive
**FB_1**	722.4	352.2	5.11	0.99	116	40	7	Positive
**FB_1**	722.4	334.2	5.11	0.99	116	44	7	Positive
**FB_2**	706.4	337.1	5.66	0.60	90	32	7	Positive
**FB_2**	706.4	319.0	5.66	0.60	90	36	7	Positive
**HT-2 Toxin**	442.2	263.2	5.30	0.96	80	8	7	Positive
**HT-2 Toxin**	442.2	197.2	5.30	0.96	80	12	7	Positive
**OTA**	404.1	358.0	5.98	1.01	102	12	7	Positive
**OTA**	404.1	238.9	5.98	1.01	102	24	7	Positive
**OTA**	404.1	220.9	5.98	1.01	102	40	7	Positive
**T-2 Toxin**	484.2	305.0	5.63	1.06	88	8	7	Positive
**T-2 Toxin**	484.2	215.0	5.63	1.06	88	16	7	Positive
**T-2 Toxin**	484.2	185.0	5.63	1.06	88	20	7	Positive
**ZEN**	317.2	273.1	6.07	1.05	130	12	7	Negative
**ZEN**	317.2	175.1	6.07	1.05	130	20	7	Negative

AFB_1: Aflatoxin B_1; AFB_2: Aflatoxin B_2; AFG_1: Aflatoxin G_1; AFG_2: Aflatoxin G_2; CIT: Citrinin; DON: Deoxynivalenol; FB_1: Fumonisin B_1; FB_2: Fumonisin B_2; OTA: Ochratoxin A; ZEN: Zearalenone

Table S2. Recovery results in multitoxin analysis

Mycotoxin Name	LOD (μg/kg)	LOQ (μg/kg)	Recovery Rate (%)	Standard Uncertainty
**DON**	29.5	95.0	91.6	0.135
**AFG_2**	0.24	0.78	96.8	0.156
**AFG_1**	0.22	0.74	92.9	0.291
**AFB_2**	0.26	0.82	94.7	0.175
**AFB_1**	0.24	0.78	95.2	0.169
**FB_1**	31.1	103	97.7	0.122
**HT-2 Toxin**	3.32	11.1	103.5	0.185
**CIT**	30.4	101	99.7	0.143
**T-2 Toxin**	3.16	10.6	102.6	0.169
**FB_2**	17.1	57.0	90.5	0.175
**OTA**	0.26	0.85	94.2	0.125
**ZEN**	2.94	9.80	94.0	0.139

AFB_1: Aflatoxin B_1; AFB_2: Aflatoxin B_2; AFG_1: Aflatoxin G_1; AFG_2: Aflatoxin G_2; CIT: Citrinin; DON: Deoxynivalenol; FB_1: Fumonisin B_1; FB_2: Fumonisin B_2; OTA: Ochratoxin A; ZEN: Zearalenone

Table S3. R^2 and regression equation

Mycotoxin	R^2	Regression Equation
**DON**	0.9966	y = 151.92x – 3103.2
**AFG_2**	0.9984	y = 1.297.9x – 245.7
**AFG_1**	0.9995	y = 3158.9x – 544.43
**AFB_2**	0.9990	y = 3586x – 28
**AFB_1**	0.9987	y = 1445.7x – 198.24
**FB_1**	0.9900	y = 2504.2x – 44501
**HT-2 Toxin**	0.9952	y = 129.57x+23.176
**CIT**	0.9945	y = 97629x – 195108
**T-2 Toxin**	0.9975	y = 2770.5x – 72452
**FB_2**	0.9915	y = 823.2x – 16704
**OTA**	0.9930	y = 278759x – 77871
**ZEN**	0.9926	y = 268.51x – 320.81

AFB_1: Aflatoxin B_1; AFB_2: Aflatoxin B_2; AFG_1: Aflatoxin G_1; AFG_2: Aflatoxin G_2; CIT: Citrinin; DON: Deoxynivalenol; FB_1: Fumonisin B_1; FB_2: Fumonisin B_2; OTA: Ochratoxin A; ZEN: Zearalenone
